# Insights into Centromere DNA Bending Revealed by the Cryo-EM Structure of the Core Centromere Binding Factor 3 with Ndc10

**DOI:** 10.1016/j.celrep.2018.06.068

**Published:** 2018-07-17

**Authors:** Wenjuan Zhang, Natalya Lukoynova, Shomon Miah, Jonathan Lucas, Cara K. Vaughan

**Affiliations:** 1 Institute of Structural and Molecular Biology, Birkbeck College, Malet Street, London WC1E 7HX, UK; 2 Institute of Structural and Molecular Biology, University College London, Gower Street, London WC1E 6BT, UK

**Keywords:** point centromere, kinetochore, CBF3, cryo-EM, Ndc10

## Abstract

The centromere binding factor 3 (CBF3) complex binds the third centromere DNA element in organisms with point centromeres, such as *S. cerevisiae*. It is an essential complex for assembly of the kinetochore in these organisms, as it facilitates genetic centromere specification and allows association of all other kinetochore components. We determined high-resolution structures of the core complex of CBF3 alone and in association with a monomeric construct of Ndc10, using cryoelectron microscopy (cryo-EM). We identify the DNA-binding site of the complex and present a model in which CBF3 induces a tight bend in centromeric DNA, thus facilitating assembly of the centromeric nucleosome.

## Highlights

• Cryo-EM studies of CBF3 reveal the core complex has a deep asymmetric channel
• The size, conservation, and charge of the channel suggest that it binds centromere DNA
• Unique insertions in the Ctf13 F box provide the binding site for one Ndc10 monomer
• The Ndc10 DNA-binding site is in plane with and perpendicular to the CBF3 channel

## Graphical Abstract

**Figure:**
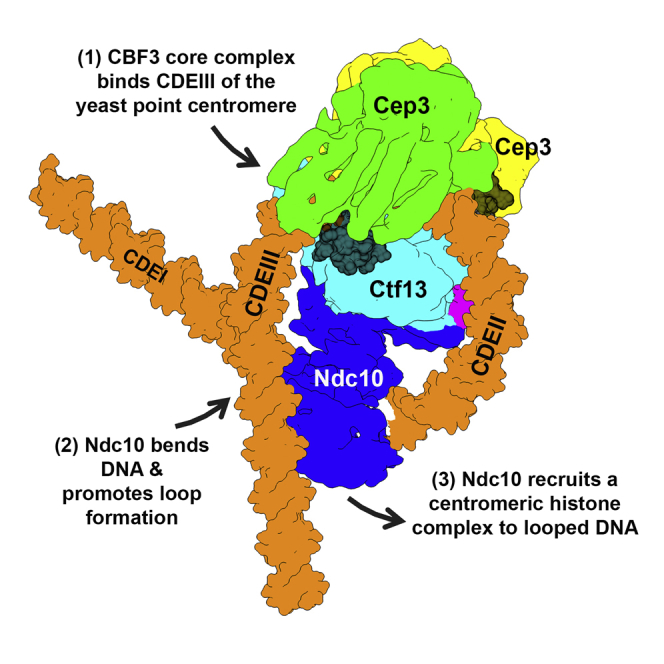

## Teaser

Zhang et al. determine two cryo-EM structures of the budding yeast centromere binding factor CBF3. These structures show that CBF3 must bend the centromere DNA element CDEIII, and this may promote looping of the centromere to accommodate a centromeric nucleosome.

## Introduction

The integrity of genetic information passed through generations relies on faithful segregation of chromosomes during mitosis. The kinetochore, a mega-Dalton protein assembly, enables this segregation by specifically associating with both the centromere (CEN) of sister chromatids and the microtubules of the mitotic spindle. Most eukaryotes have regional CENs with unique satellite repeats that vary in length but whose arrangement is largely conserved between chromosomes despite being unconserved in sequence. Regional CENs, which can be up to 5 Mb in length in humans, are specified epigenomically by the presence of an essential centromeric histone H3 variant, CENP-A, which is found at CEN DNA, where it is interspersed with canonical nucleosomes (Verdaasdonk and Bloom, 2011).

In contrast, budding yeasts, including *S. cerevisiae*, have evolved point CENs, comprising three CEN DNA elements (CDEs), typically of ∼125 bp. CDEI (8 bp) and CDEIII (∼26 bp) are relatively conserved and border CDEII (78–86 bp), which, although largely unconserved at a base level, is characterized by generally greater than 90% A-T content (Fitzgerald-Hayes et al., 1982). Point CENs evolved from an ancestor with an epigenomically specified CEN (Malik and Henikoff, 2009), and this evolutionary transition introduced genetic specification of the CEN while retaining aspects of epigenomic specification, in particular the essential presence of the CENP-A homolog Cse4.

Unique to these organisms, the CEN binding factor 3 (CBF3) complex provides a physical link between the genetic and epigenomic mechanisms of CEN specification. It associates specifically with CDEIII (Lechner and Carbon, 1991) and is responsible for deposition of Cse4, through a direct interaction with the centromeric histone chaperone Scm3/HJURP (Camahort et al., 2007). Its epigenomic role is emphasized by the observation that a synthetic kinetochore can be assembled in the absence of CDEs, provided functional CBF3 is available to stabilize Cse4 incorporation at the CEN (Ho et al., 2014).

CBF3 comprises two homodimers, of Cep3 and Ndc10, and a Ctf13-Skp1 heterodimer. Cep3 provides sequence specificity through binuclear zinc-cluster (Zn_2_Cys_6_) domains homologous to those found in GAL4-like fungal transcription factors (Lechner, 1994, MacPherson et al., 2006). These domains bind a pseudo-TGT/CCG palindrome in CDEIII (Espelin et al., 1997). Ndc10 contributes both non-specific DNA binding (Cho and Harrison, 2011, Espelin et al., 1997) and association with Scm3/HJURP (Camahort et al., 2007). The Skp1-Ctf13 heterodimer interacts with Cep3, Ndc10, and CDEIII at a completely conserved G, centrally positioned between the TGT/CCG half-sites (Espelin et al., 1997).

The molecular mechanism of CBF3 association with CDEIII is unknown, despite a wealth of genetic, biochemical, and structural data (Bellizzi et al., 2007, Cho and Harrison, 2011, Leber et al., 2018, Perriches and Singleton, 2012, Purvis and Singleton, 2008), because of an absence of atomic-resolution structural information for the CBF3 complex as a whole.

Herein we present cryoelectron microscopy (cryo-EM) structures of the CBF3 core complex (CBF3CCΔN) alone at atomic resolution and in complex with a monomeric construct of Ndc10 (CBF3CCΔN + Ndc10 D1-2). The core complex forms a deep channel that is both highly charged and strongly conserved and is perfectly sized to accommodate DNA. The structure of CBF3CCΔN + Ndc10 D1-2 identifies the Ndc10 D1-2 interface on the core complex and indicates a second DNA-binding interface that is in plane with, and perpendicular to, the channel of the core. Combining our *in vitro* experiments, which identify the channel as the likely site of DNA association with the core complex, and existing structural data for the DNA-binding site of Ndc10 D1-2, we propose a model that accounts for previously published genetic, biochemical, and biophysical experiments and implicates the core complex as well as Ndc10 in bending DNA to accommodate the centromeric nucleosome.

## Results

### Cryo-EM Studies of CBF3

**Figure 1:**
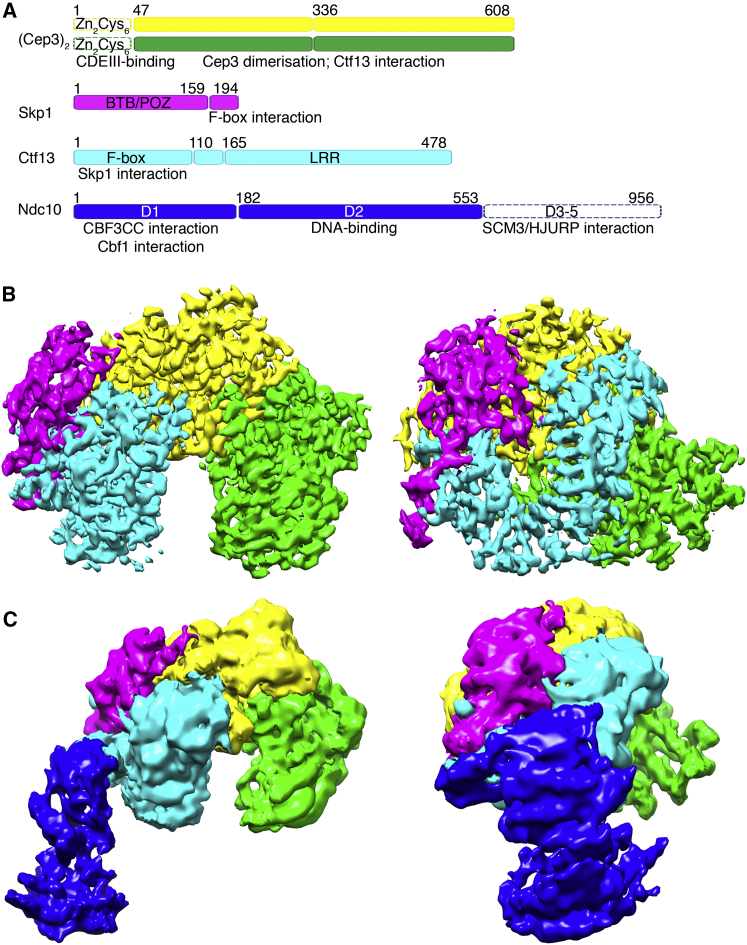
Cryo-EM Reconstructions of CBF3 (A) Components of CBF3, annotated with domain boundaries and architecture. Known binding partners are indicated below the relevant domains. Domains not included in the constructs used for structure determination are hatched. (B and C) Orthogonal views of CBF3CCΔN (B) and CBF3CCΔN + Ndc10 D1-2 (C) colored by protein. See also Figures S1 and S2 and Table S1.

Dissection of the assembly and turnover of CBF3 *in vivo* indicated that Cep3 and Ctf13 associate early in the CBF3 assembly pathway, whereas association with Ndc10 occurred at much later time points (Rodrigo-Brenni et al., 2004). These results were in agreement with earlier *in vitro* reconstitution experiments (Russell et al., 1999); therefore we expressed and purified a CBF3CC comprising the Ctf13-Skp1 heterodimer and the Cep3 homodimer (Figure 1A). Full-length Cep3 rendered CBF3CC relatively unstable, but the complex with an N-terminally truncated Cep3, in which the Zn_2_Cys_6_ domains were missing, yielded a 220 kDa complex (CBF3CCΔN) that was purified to homogeneity and was suitable for cryo-EM studies (Figures S1A and S1B). Two-dimensional (2D) classification of motion-corrected cryo-EM images generated class averages with clear secondary structural details (Figure S1C) that enabled *de novo* reconstruction of a three-dimensional (3D) map (Figure S1D). After refinement, the final map was calculated from 187,606 particles, with a resolution of 3.7 Å judged using the Fourier shell correlation (FSC) gold standard method (Figures S1E–S1H; Table S1). The CBF3CCΔN map has a horseshoe shape with a deep central channel (Figure 1B).

Ndc10 is the remaining and largest component of the CBF3 complex (110 kDa) which, like Cep3, is dimeric. Current data suggest that each of its five domains contributes different functions (Cho and Harrison, 2011): domain 1 associates with both the CBF3 core and Cbf1, a non-essential CDEI-binding protein; domain 2 provides the principal DNA-binding interface; domain 3 contributes dimerization; and domains 4 and 5 are responsible for recruitment of Scm3/HJURP.

In order to identify the binding site for Ndc10 on CBF3CCΔN we assembled a complex of CBF3CCΔN bound to a monomeric construct of Ndc10 comprising domains 1 and 2 (Ndc10 D1-2). After co-elution by size exclusion chromatography, this complex was suitable for cryo-EM studies. Two-dimensional classification revealed high-resolution classes in which secondary structural elements could be identified, and these data generated a 4.2 Å map (Figures 1C and S2A–S2E; Table S1).

### Atomic Model of CBF3CCΔN

**Figure 2:**
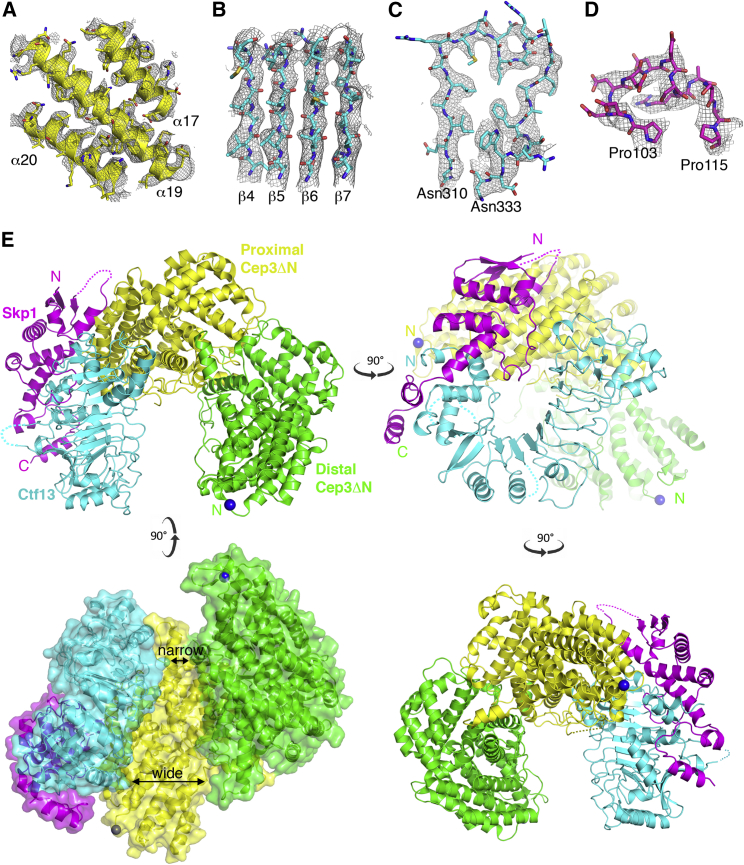
Atomic Model of CBF3CCΔN (A–D) Representative electron density for helices from Cep3ΔN (A), four β strands of the LRR β sheet from Ctf13 (B), LRR4 from Ctf13 (C), and a loop in Skp1 (D). (E) The structure of CBF3CCΔN showing four views each related by ∼90°. The N termini of the Cep3ΔN homodimer are shown as blue balls. See also Figures S3–S5.

Secondary structure details and side chains were clearly visible throughout the CBF3CCΔN map (Figures 2A–2D), enabling an atomic model for the core complex to be built and refined (Figure 2E; Table S2). The Cep3 homodimer was readily recognized, facilitated by its largely helical structure and the identification of an approximately two-fold axis, which could also be seen in the 2D classes (Figure S1C). The 2.5 Å crystal structure of the Cep3ΔN homodimer (Purvis and Singleton, 2008) was fit in the map as a rigid body, and this structure is essentially unchanged after refinement with a final Cα RSMD of 1.1 Å. The consistently high quality of the remaining density, assigned to the Skp1-Ctf13 heterodimer, has highlighted significant structural and chemical details that were not visible in the previously published poly-alanine model of Ctf13 within CBF3CC (Leber et al., 2018).

The most striking secondary structural feature of this model is an eight-stranded parallel β sheet. This is part of a larger solenoid structure, corresponding to the predicted leucine-rich repeat (LRR) fold of Ctf13, and comprises eight LRR motifs, rather than seven LRRs identified previously (Leber et al., 2018). The LRRs are flanked at each end by additional domains (Figures 2E and S3A). The N-terminal BTB/POZ domain of yeast Skp1 (Orlicky et al., 2003) could be fit as a rigid body into one flanking domain. As previously observed (Leber et al., 2018), the CBF3 Skp1-Fbox structure is atypical, with the Skp1 helices α7 and α8 reoriented by 86° and 60°, respectively (Figures S3B, S3C, S5A, and S5B). Our model also reveals a unique linker subdomain between the F box and the LRRs, comprising a three-stranded anti-parallel β sheet and a long α-helix (Figure S3A). The majority of the F box is well conserved between Saccharomycetaceae, so this atypical structure is likely to be found in all Skp1-Ctf13 homologs (Figure S4A). In addition, there is an ∼50 amino acid insertion between α2 and α3 of the F box for which there is no electron density, despite the latter half having significant sequence conservation (see further results below).

The remaining flanking density is an α-β subdomain that decorates the C-terminal end of the Ctf13 LRR, formed by insertions within the last 3 LRRs of Ctf13. This subdomain contacts and stabilizes a typically disordered acidic loop of Skp1 such that the Skp1-Ctf13 heterodimer forms a toroidal structure in which both the N-terminal F box and the C-terminal α-β LRR-insertion domain of Ctf13 associate with either end of Skp1 (Figures 2E and S5C). Other LRR-containing F box proteins form toroids, notably both the TIR1 and COI1 plant hormone receptors (Sheard et al., 2010, Tan et al., 2007), but in these cases the toroid is formed by the LRR domain alone, with Skp1 and the LRR domains almost perpendicular to each other. In contrast, for Skp1-Ctf13, the unusual Skp1-F box interaction forces both Skp1 and Ctf13 into the same plane (Figures 2E and S3D).

The Ctf13-Skp1 heterodimer forms the left side of the horseshoe and makes extensive contacts with the base, formed by the “proximal” monomer of the Cep3 homodimer (Figures 2E and S5D–S5F). This interface includes density for part of a loop in Cep3, from residues 330–339, not previously visible in the crystal structures. The “distal” monomer of the Cep3 dimer forms the remaining side of the horseshoe (Figure 2E). This arrangement positions the truncated Zn_2_Cys_6_ domains of (Cep3ΔN)_2_ at opposite ends of the channel created by the horseshoe. The position of Ctf13 relative to (Cep3ΔN)_2_ results in the channel being considerably narrower next to the N terminus of the distal Cep3ΔN monomer (Figure 2E).

### CBF3CCΔN Channel Is the Putative Binding Site for CDEIII

**Figure 3:**
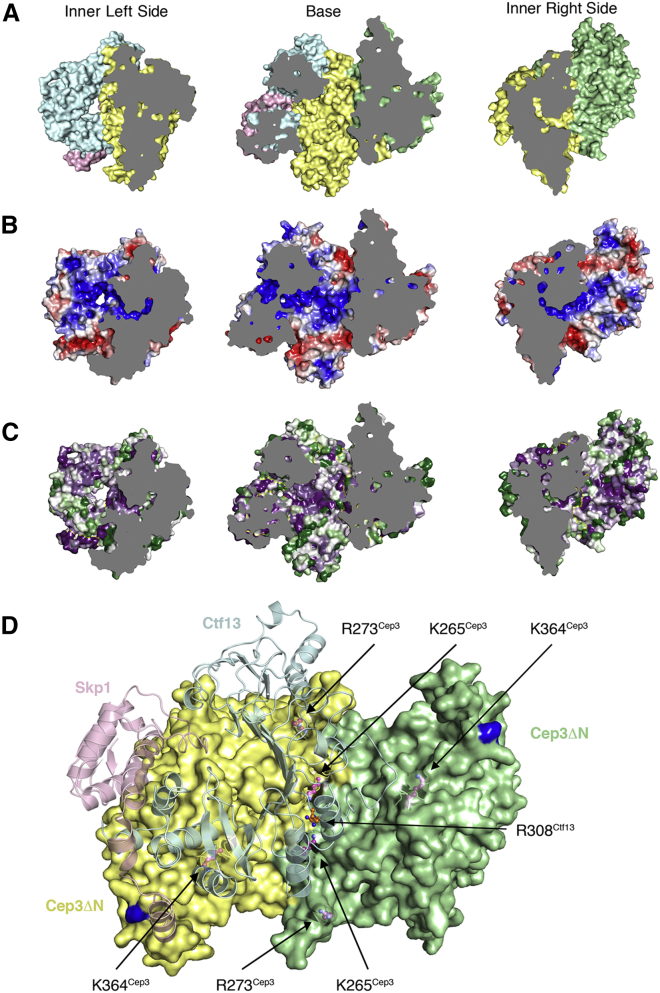
The CBF3CCΔN Channel Is the Putative Binding Site for CDEIII Views of each side of the inner surface of the channel colored by protein (color as in D) (A), electrostatic potential (from −5 [red] to +5 [blue] TeV) (B), and conservation (purple, white, and green denote high, medium, and low conservation, respectively) (C). (D) View down the two-fold axis of the Cep3ΔN homodimer. R308^Ctf13^ lies directly along the two-fold axis (orange ball and stick). Conserved basic residues in Cep3 are highlighted in magenta ball and stick.

Ctf13 and Cep3 line the channel with basic residues that are strongly conserved between Saccharomycetaceae (Figures 3A–3C). In Ctf13, a series of arginine and lysine residues extend like fingers from the inter-LRR turns of LRRs 1–6 into this channel (Figure S3E). LRR3 projects two neighboring arginines, Arg307 and Arg308, and the latter is positioned directly along the two-fold axis of (Cep3ΔN)_2_, mid-way between the truncated (Cep3ΔN)_2_ N termini (Figure 3D). Additional conserved basic residues from Cep3ΔN extend toward the channel from each Cep3 protomer, including Lys265, Arg273, and Lys364.

**Figure 4:**
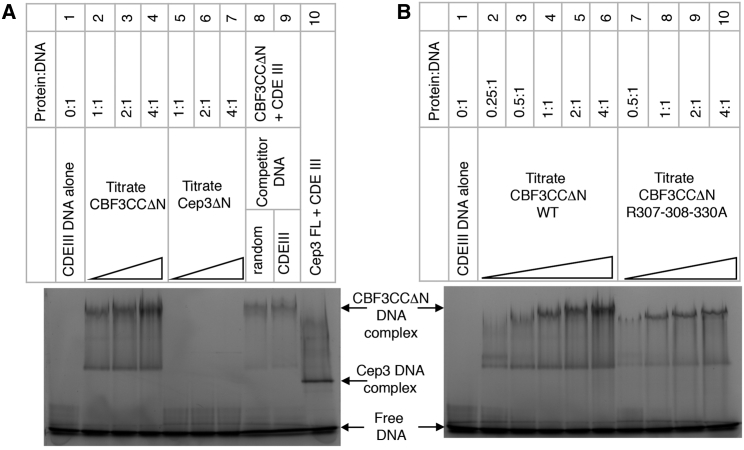
EMSAs for CBF3CCΔN (A) Fluorescently labeled CDEIII DNA (1.6 μM) and a titration of CBF3CCΔN (lanes 2–4) or Cep3ΔN (lanes 5–7) or 6.4 μM of full-length Cep3 (lane 10). Lanes 8 and 9 show competition of CBF3CCΔN binding with 80 μM unlabeled probe. (B) Fluorescently labeled CDEIII DNA (1.6 μM) with a titration of CBF3CCΔN (lanes 2–6) and CBF3CCΔN with Ctf13 mutations R307A-R308A-R330A. See also Figure S6.

The charge and conservation within the channel, and the striking relative orientation of the arginine residues of neighboring LRRs in Ctf13, suggested that the channel may provide the binding site for CEN DNA, with these residues potentially contributing direct interactions with CDEIII. In order to test this model, we carried out electrophoretic mobility shift assays. Full-length Cep3 alone binds CDEIII tightly, whereas Cep3ΔN, with truncated Zn_2_Cys_6_ domains, does not bind CDEIII DNA, consistent with previous observations (Figure 4A, lanes 5–7 and 10) (Purvis and Singleton, 2008). In contrast, CBF3CCΔN shows a DNA gel shift (Figure 4A, lanes 2–4 and 5–7), indicating that association with the Skp1-Ctf13 heterodimer significantly enhances the affinity of Cep3ΔN for CDEIII DNA. This observed shift does not require dephosphorylation of Skp1 (Leber et al., 2018). Labeled CDEIII DNA could be competed with either unlabeled CDEIII DNA or a random DNA duplex of equal length (Figure 4A, lanes 8 and 9) indicating that the association is not sequence specific. Our *in vitro* data therefore provide conclusive evidence that the Zn_2_Cys_6_ domains are not required for the association of the CBF3CC complex with DNA. However, the Zn_2_Cys_6_ domains are the sole determinants of sequence specificity, as the remaining component of the CBF3, Ndc10, has also been shown to contribute affinity not but specificity to the CDEIII association (Cho and Harrison, 2011).

We tested the contribution of the highly conserved arginines 307, 308, and 330 from Ctf13 and lysines 265 and 364 from Cep3 to this interaction. The triple mutation to alanine in Ctf13 reduces association of CBF3CCΔN with DNA (Figure 4B). This effect is amplified by further mutation of Lys265 and Lys364 to alanine in Cep3 (Figure S6A). However, we note that although the mutant complex purifies in a manner consistent with a fully folded complex, it is destabilized relative to the wild-type (WT) and Ctf13-triple-alanine mutant (Figure S6B). Considered together, these data are consistent with a model in which basic residues lining the channel contribute affinity to DNA binding.

### Ndc10 Associates with the Atypical F Box of Ctf13

**Figure 5:**
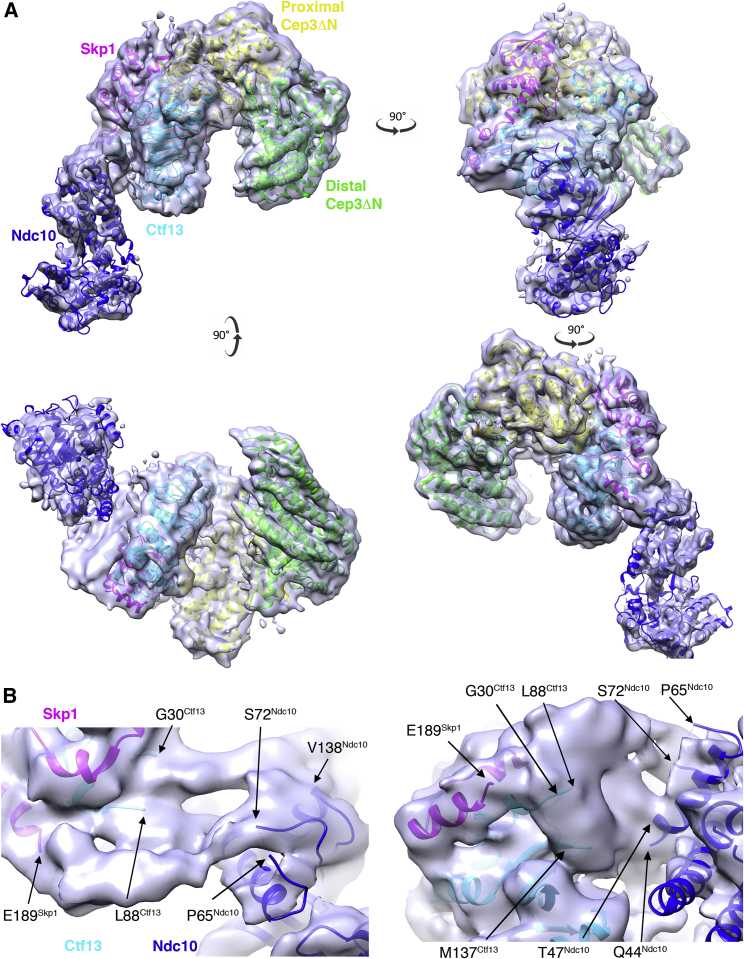
Ndc10 Associates with the Atypical F Box of Ctf13 (A) Views of the composite map for the CBF3CCΔN (gray) + Ndc10 D1-2 (blue) with the model for CBF3CCΔN and the crystal structure of *S. cerevisiae* Ndc10 fitted. (B) Two close-up views of the interface. Ordered residues from the atomic models that border this interface are highlighted. See also Figure S7.

Our map of the complex between CBF3CCΔN and Ndc10 D1-2 has excellent density for the core complex and lower resolution for Ndc10, suggesting flexibility between the two. Focused refinement of Ndc10 D1-2 yielded a second map with improved density for Ndc10 (Figures S2E and S2F; Table S1). Superposition of the common parts of these two maps yielded a composite map, which combined the high-resolution core from the original map and the improved Ndc10 density from the second map. The atomic model of CBF3CCΔN fits readily into our composite map, and the remaining density accommodates the crystal structure of *S. cerevisiae* Ndc10 D1-2 (Perriches and Singleton, 2012) (Figure 5A). Closer inspection reveals that the majority of the interface is formed from structural elements that are disordered in the structures of the isolated components. Ndc10 binds to the F box of Ctf13 on the outside of the channel, and two unique structural elements of this atypical F box are responsible for the interaction. Specifically, within Ctf13, the long insertion between α2 and α3 (approximately amino acids 30–90), which is predicted to be helical and contains stretches of conservation particularly through residues 70–90 (Figure S4), and a turn between β2 and β3 of the antiparallel β sheet, locate to density at the interface with Ndc10 D1-2. The interface on Ndc10 is mediated primarily by residues at its N terminus (before Gln 44) which are also predicted to be helical, with minor contributions from the C-terminal end of α3 (Val138) and a disordered loop between α1 and α2 (Pro65–Ser72) (Figures 5B and S7A). The C-terminal residues of Skp1 (Glu189–Arg194) are also located at the interface. Together these structural elements form a hinge between the core complex and Ndc10 leading to lower resolution for this region of the cryo-EM map.

The resulting complex associates tightly with DNA relative to the binding by CBF3CCΔN alone (Figure S7B).

## Discussion

### Structural Homology of Ctf13 Suggests an Evolutionary Link to Epigenomic Modifications of the Point CEN

The consistently high resolution of our map for CBF3CCΔN allowed an atomic model of Ctf13 to be built. A search of PDBeFold and Dali identifies the histone demethylase KDM2B as the closest known F box-containing structural homolog of Ctf13 (Figures S3F–S3G).

KDM2B is a human lysine demethylase from the JHDM1 family containing the Jumonji (JmjC) domain. In general, the members of this family are responsible for inducing transcriptional silencing through demethylation of H3K36. In lower eukaryotes, family members, such as Jhd1 in *S. cerevisiae*, comprise only the histone lysine demethylation domain, while in higher eukaryotes they have several additional domains that detect or alter epigenomic states (Klose et al., 2006). In humans, these include a zinc finger domain that recognizes methylated DNA and an F box and LRR domain that can recruit SCF E3 ubiquitin ligase activity (Han et al., 2016, Wong et al., 2016).

CENs have been subject to rapid recent evolutionary change, accounting for the wide diversity in CEN sequences among species. Evidence suggests that the point CENs of Saccharomycetaceae evolved from an ancestor with a regional CEN, and the CBF3 complex co-evolved to meet the requirements of genetic specification (Malik and Henikoff, 2009). The structural homology between Ctf13 and KDM2B may indicate an evolutionary path for the Skp1-Ctf13 component of CBF3 in budding yeasts, involving the partition of a KDM2B-like chromatin-associated enzyme in a common ancestor into two independent genes: the JHMD1 family member Jhd1 and point CEN-associated Ctf13. Whether there is a genetic or physical link between the two resultant genes in extant budding yeasts remains to be determined. Jhd1 has been shown to counter Set2 methylation (Fang et al., 2007), which is associated with RNA polymerase (pol) II transcription (Kim and Buratowski, 2007, Kwon and Ahn, 2011, Sein et al., 2015) and suppression of histone exchange (Venkatesh et al., 2012), but to date, no centromeric function has been attributed. In contrast, in *S. pombe*, the KDM2B homolog Epe1 contributes to CEN function through regulating heterochromatin boundaries (Trewick et al., 2007). Epe1 may therefore represent an evolutionary intermediate, as it functions to define a regional CEN, but like Jhd1, it is a “minimal” JHDM1 family member, comprising only the JmjC domain without additional targeting domains.

Although structural homology alone is not sufficient to indicate functional analogy, it is tempting to speculate that the structural homology of Ctf13 with KDM2B may indicate a molecular link between Ctf13 and epigenomic specification of point CENs. Such a link has already been shown to exist for Ndc10, as it associates with the histone chaperone Scm3/HJURP, thereby recruiting the centromeric histone Cse4 (CenH3) to the point CEN. Ctf13 may therefore also contribute a currently uncharacterized function in epigenomic specification of the budding yeast point CEN.

### Model for CBF3 Association with CDEIII

**Figure 6:**
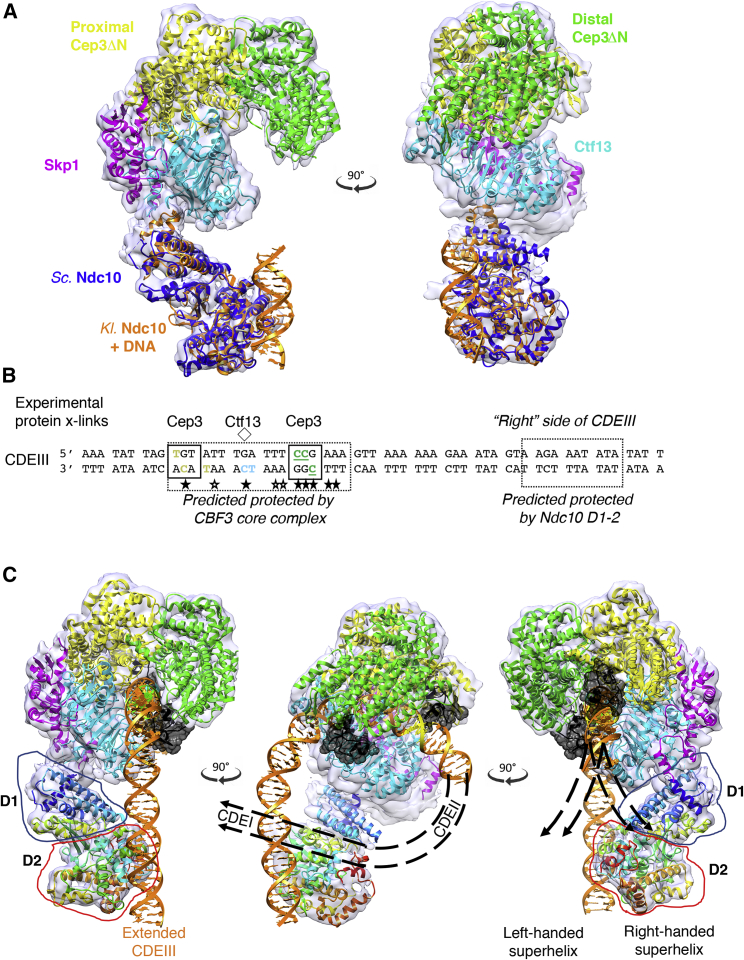
Model for CBF3 Association with CDEIII (A) Two views of a superposition of DNA-bound *K. lactis* Ndc10 D1-2 (PDB: 3SQI) (orange) within the *S. cerevisiae* model. (B) Sequence of CEN3 CDEIII. Half-sites of the pseudo-palindrome are boxed. The pseudo-dyad axis is marked with a diamond. Completely conserved and strongly conserved bases are indicated with filled and empty stars, respectively. Bases that crosslink with Cep3 or Ctf13 are highlighted in color; bases whose labeling interferes with CBF3 binding are underlined. Bases that are predicted in our model to interact with either the CBF3 core or Ndc10 D1-2 are enclosed in a dashed box. (C) Model of the complex bound to extended CDEIII DNA. Ndc10 D1-2 is colored in rainbow, and the individual domains are framed. The CCG and TGT half-sites are colored green and yellow, respectively. The modeled Cep3 Zn_2_Cys_6_ domains are shown in black (PDB: 1D66) on each half-site. Possible predicted paths of the CDEII and CDEI DNA following a left- or right-handed superhelix are shown. See also Figure S8.

Our electrophoretic mobility shift assay (EMSA) results support a model in which CDEIII DNA binds in the CBF3CCΔN channel, and consistent with this the diameter of the channel accommodates a modeled DNA duplex (Figure S8A). Superposition of the crystal structure of DNA-bound Ndc10 D1-2 from the budding yeast *Kluyveromyces lactis* (Cho and Harrison, 2011) onto our model indicates that the DNA-binding site of Ndc10 is coplanar with, and approximately perpendicular to, the CBF3CCΔN channel (Figure 6A). Therefore, to satisfy both the core and Ndc10 DNA-binding sites, the DNA must bend significantly.

Previous crosslinking data identified crosslinks between Ctf13 and the completely conserved cytosine at the pseudo-dyad axis, and its neighboring 3′ thymine, on the bottom strand of CEN3 CDEIII (Espelin et al., 1997) (Figure 6B). Alignment of the pseudo-dyad axis of modeled CEN3 CDEIII DNA with the two-fold axis of Cep3 places the central cytosine in line with the conserved Arg308 of Ctf13. If the DNA is then oriented such that the most conserved surface of Ctf13 aligns with the conserved “right-hand” end of CDEIII, the CCG half-site is placed at the narrow point of the channel (Figure S8A). There is strong sequence conservation between the Zn_2_Cys_6_ domains of Cep3 and fungal transcription factors, including between residues that contribute to half-site recognition (Figure S8D). When the Zn_2_Cys_6_ of Cep3 is modeled using the prototypical GAL4 crystal structure (Marmorstein et al., 1992), the narrow gap perfectly accommodates the Zn_2_Cys_6_ domain and orients its C-terminal end toward the N-terminal end of Cep3ΔN (Figure S8B). This superposition packs the Zn_2_Cys_6_ domain against a strongly conserved loop between LRR motifs 3 and 4 (Figure S4).

The equivalent superposition at the other half-site is sterically incompatible with linear DNA (Figure S8C). However, the CEN DNA of budding yeast, comprising CDEI–CDEIII, forms a centromeric nucleosome (Furuyama and Biggins, 2007) with atypical (Dalal et al., 2007, Dimitriadis et al., 2010, Mizuguchi et al., 2007, Zhang et al., 2012), cell cycle-dependent histone composition (Bui et al., 2012, Shivaraju et al., 2012). Modeling a bend in CDEIII using nucleosome parameters of DNA roll, slide, and twist (Tolstorukov et al., 2007) allows the second Zn_2_Cys_6_ domain to be readily accommodated in a TGT-binding conformation (Figure 6C). This model places the conserved CCG and TGT half-sites in very different environments: the former is buried by Ctf13 and Cep3 at the narrow end of the channel, whereas the latter is solvent accessible at the open end of the channel. This structural asymmetry within the core complex, in particular the increasing width of the DNA-binding channel, and the modeled encapsulation of the CCG half-site by one Zn_2_Cys_6_ domain, accounts for observed asymmetric chromosome nondisjunction rates seen when CDEIII is altered: mutations centered around the CCG half-site cause rates of chromosome loss up to 2 orders of magnitude greater than mutations of the TGT (Hegemann et al., 1988). Similarly, labeling of the CCG bases is observed to cause significant loss of association with CBF3 compared with the TGT half-site (Espelin et al., 1997), and genetic results identify the CCG triplet as the only bases within the CEN whose substitution cannot be supported in *S. cerevisiae* (Cumberledge and Carbon, 1987, Gaudet and Fitzgerald-Hayes, 1987, Jehn et al., 1991, McGrew et al., 1986, Ng and Carbon, 1987, Niedenthal et al., 1991). Leber et al. (2018) identified a binding site for the TGT-proximal Zn_2_Cys_6_ domain between Ctf13 and Cep3 in a manner that would not allow binding to the TGT half-site. Because this location is not compatible with the observation of crosslinks between the TGT half-site and Cep3 (Espelin et al., 1997), it may be possible that this is an inactive conformation and that the presence of DNA and/or dephosphorylation may unlock it from this docked position.

Linear extension of the modeled DNA beyond CDEIII to incorporate bases to the “right” of the CEN DNA places bases approximately 21–30 bp downstream of the CCG half-site at the Ndc10 DNA-binding site (Figures 6B and 6C). This is in agreement with Ndc10 crosslinking results indicating that Ndc10 forms sequence-specific interactions with a series of thymines to the “right” side of CDEIII (Espelin et al., 1997). As a whole, the complex encompasses a stretch of approximately 50 bp of CDEIII, remarkably consistent with the established 56 bp DNaseI footprint of CBF3 (Lechner and Carbon, 1991).

The centromeric nucleosome of *S. cerevisiae* protects ∼121 bp DNA (Cole et al., 2011, Krassovsky et al., 2012), the majority of which is AT-rich CDEII. Independent experimental observations suggest that the CEN DNA is wound in a right-handed manner and that this is dependent on the sequences within CDEII and CDEIII (Díaz-Ingelmo et al., 2015, Furuyama and Henikoff, 2009), contrary to the left-handed DNA supercoiling in canonical nucleosomes. Similar observations in higher eukaryotes suggest that this is not unique to organisms with point CENs (Furuyama and Henikoff, 2009).

Our Ndc10 construct is monomeric, so the position of the second Ndc10 protomer is unknown. Nonetheless, the C terminus of our construct is oriented toward the CDEII-proximal side of the modeled DNA (Figure 6C), suggesting that second Ndc10 protomer may also orient toward CDEII. Such a location is consistent with gel shift data indicating that Ndc10 associates specifically with CDEII (Espelin et al., 2003) and biophysical and biochemical experiments showing the Ndc10 dimer binds two separate segments of DNA (Cho and Harrison, 2011). In this scenario, the curvature of CDEIII induced by the core complex and the CDEIII-bound Ndc10 protomer is further enhanced at CDEII by the second Ndc10 protomer, in agreement with topology analysis of plasmids containing CEN DNA in budding yeast (Díaz-Ingelmo et al., 2015) and thus accounting for the observed looping of CEN DNA (Pietrasanta et al., 1999).

In our model, both a right-handed and a left-handed DNA supercoil can be accommodated (Figure 6C). A right-handed superhelical path would position CDEI close to Ndc10 D1, consistent with data indicating that the CDEI-binding protein Cbf1 associates directly with Ndc10 D1 (Cho and Harrison, 2011). Further structural data that definitively locate the second Ndc10 protomer will be required to resolve this ambiguity.

Our structures of the CBF3CCΔN alone and in complex with Ndc10 D1-2 and the resulting model of CDEIII binding explain a wealth of data published over several decades, including genetic analyses (Cumberledge and Carbon, 1987, Gaudet and Fitzgerald-Hayes, 1987, Hegemann et al., 1988, Jehn et al., 1991, McGrew et al., 1986, Ng and Carbon, 1987, Niedenthal et al., 1991), crosslinking (Espelin et al., 1997, Espelin et al., 2003), and footprinting data for CBF3 (Lechner and Carbon, 1991). Our structures indicate the path of DNA is strongly curved at CDEIII and probably looped at CDEII by the second protomer of Ndc10, consistent with atomic force microscopy (AFM) experiments that show shortening and bending of CEN DNA when bound to CBF3 (Pietrasanta et al., 1999).

## Experimental Procedures

### Protein Expression and Purification

Our experiments showed that the co-expression of Sgt1 helps the formation and stability of CBF3CCΔN; therefore we cloned and co-expressed Sgt1 with CBF3CCΔN components. The SGT1 gene with a C-terminal Strep tag was cloned into a modified pRS424 vector containing the CTF13 gene with a C-terminal CBP tag. The SKP1 gene was cloned into the modified pRS426 vector containing CEP3 (residues 47–608) with an N-terminal His tag. Mutants were generated by site-directed mutagenesis PCR. Both plasmids were co-transformed into *S. cerevisiae* yeast strain BCY123 (MATα pep4::HIS3 prb1::LEU2 bar1::HIS6 lys2::GAL1/10GAL4 can1 ade2 trp1 ura3 his3 leu23,112) by using -Trp, -Ura selection plates (yeast nitrogen base, Trp and Ura dropout mix [Formedium], 55 mg/mL adenine, 55 mg/mL L-tyrosine, and 2% glucose). Expression of the complex and mutants was performed in BCY123. The cells were pre-cultured in selective media and then inoculated into non-selective medium with 2% raffinose to a starting optical density (OD) of 0.25. Expression was induced with 2% galactose for 16 hr at an OD of 0.9–1.0. Pelleted cells were resuspended in lysis buffer A (50 mM Tris [pH 8.0], 500 mM NaCl, 2 mM Mg acetate, 2 mM imidazole, 4 mM CaCl_2_, and 0.2% Igepal CA-630) supplemented with cOmplete EDTA-free Protease Inhibitor Cocktail (Roche) and frozen as pellets in liquid N_2_. The pellets were lysed using a freezer miller (SPEX Sample Prep). The complex was purified by calmodulin resin and eluted by buffer B (10 mM Tris [pH 8.0], 500 mM NaCl, 1 mM Mg acetate, 1 mM imidazole, 4 mM EGTA, and 2 mM DTT). Purified fractions were loaded onto a His-Trap column (GE Healthcare) pre-equilibrated with buffer C (20 mM Tris, 500 mM NaCl, 20 mM imidazole [pH 8.0], and 10 mM β-mercaptoethanol). The eluted protein was loaded onto a Mono Q column (GE Healthcare) after dilution with low-salt buffer D (20 mM Tris [pH 8.0], 100 mM NaCl, 2 mM DTT, 5 mM EDTA, and 10% glycerol). The complex fractions were pooled and concentrated before loading onto a Superdex 200 5/150 GL (GE Healthcare) pre-equilibrated with S200 buffer (15 mM Tris [pH 8.0], 200 mM NaCl, and 2 mM DTT). The peak fraction was used to make EM grids.

Cep3FL with an N-terminal His-tag was expressed in *S. cerevisiae* using the same system as above and was purified using Ni-affinity chromatography, using the His-Trap, ion exchange, and size exclusion steps described above.

Domains 1 and 2 (residues 1–554) of NDC10 were cloned from yeast genomic DNA into the pRS426 vector with a C-terminal Strep tag. The (Ndc10 D1-2 pRS426) was co-transformed with an empty pRS424 plasmid to express the Ndc10 D1-2 protein. The protein was purified by StrepTrap HP column (GE Healthcare) pre-equilibrated with Strep binding buffer (20 mM Tris-HCl [pH 8.0], 500 mM NaCl, 1 mM EDTA, and 3 mM DTT) and then eluted in Strep elution buffer (binding buffer + 3 mM d-desthiobiotin). Fractions containing the desired protein were pooled and purified further by Mono Q column and Superdex 200 5/150 GL. Purified CBF3CCΔN and Ndc10 D1-2 were mixed at 1:1 molar ratio and incubated at 4°C for 1 hr. The mixture was loaded onto Superose 6 Increase 3.2/300 (GE Healthcare). The peak fraction was used to make EM grids.

### Cryo-EM

The samples (0.12 and 0.24 mg/ml for the first and second datasets of CBF3CCΔN and 0.15 mg/mL for CBF3CCΔN + Ndc10 D1-2) were applied to glow-discharged UltrAuFoil 1.2/1.3 300 mesh grids (Quantifoil Micro Tools). Cryo-EM data were acquired at the Electron Bio-Imaging Centre (eBIC) on a FEI Titan Krios at 300 keV (Thermo Fisher Scientific), equipped with a post Quantum energy filter K2 Summit direct detector (Gatan). Data collection was automatically carried out using EPU software (Thermo Fisher Scientific) at a magnification of 47,170 (1.06 Å pixel^−1^) to record 1,236 videos with a defocus range of −1.6 to −3.6 μm for the first CBF3CCΔN dataset and 1,101 videos with a defocus range of −1.0 to −4.0 μm for the second CBF3CCΔN dataset. The total exposure time of 10 s fractionated into 25 frames with a total dose of 46 e^−^ Å^−2^ for first dataset and the total exposure time of 15 s fractionated into 40 frames with a total dose of 60.9 e^−^ Å^−2^ for second dataset. Videos were aligned using MotionCor2 (Zheng et al., 2017).

An initial dataset of the complex of CBF3CCΔN + Ndc10 D1-2 was collected in-house on a Tecnai Polara operating at 300 keV, recording at 1.39 Å pixel^−1^ on a post Quantum energy filter K2 Summit direct detector operating in counting mode. The total dose was 54 e^−^ Å^−2^ for 18 s and 60 frames with a defocus range of −1.5 to −4.0 μm. The final dataset for this complex, comprising 2,003 movies, was collected at eBIC on a Titan Krios microscope using the same parameters as CBF3CCΔN, except the total dose was 50 e^−^ Å^−2^ and the defocus range was −1.5 to −3.5 μm.

### Image Processing

Contrast transfer function (CTF) parameters were estimated using CTFFIND4 (Rohou and Grigorieff, 2015), and CTF correction and following image processing were performed using RELION 2.0 (Kimanius et al., 2016), unless otherwise noted. Resolution is reported using the gold-standard FSC (0.143 criterion) as described (Rosenthal and Henderson, 2003, Scheres and Chen, 2012), and temperature factors were determined and applied automatically in RELION 2.0. For CBF3CCΔN, a subset of the initial dataset was picked using an automatically generated Gaussian reference by Gautomatch (Urnavicius et al., 2015), extracted using a 200^2^ pixel box, and then subjected to reference-free 2D classification. Some of resulting 2D class averages representing different views were selected to be low-pass-filtered to 25 Å and used as references for further automatic particle picking of the initial dataset. The automatically picked particles were screened manually, followed by reference-free 2D classification, which yielded 69,392 particles for subsequent processing. An ovoid generated with SPIDER (Shaikh et al., 2008) was used as an initial model for 3D classification. The best 3D class was used to perform a 3D auto-refinement against all the good particles, resulting in a 4.9 Å map. After substitution of the particles contributing to this map by re-extraction from dose-weighted images calculated by MotionCor2, a further 3D auto-refinement provided a reconstruction at 4.7 Å overall resolution. A total of 212,724 particles from the second dataset were picked from dose-weighted images and selected for further processing after reference-free 2D classification. After joining the two datasets, 282,116 particles were input to 3D classification using an initial 3D reference obtained by low-pass filtering (50 Å) the 4.7 Å map. After 3D classification, the best class, with 187,606 particles, was subjected to 3D auto-refinement. This resulted in a reconstruction of 4.1 Å. The map was post-processed by RELION and sharpened by a negative B factor using an automated procedure, resulting in a 3.7 Å reconstruction.

For CBF3CCΔN + Ndc10 D1-2, particles were picked automatically from the initial Polara dataset by Gautomatch (Urnavicius et al., 2015), extracted using a 152^2^ pixel box, and subjected to reference-free 2D classification, yielding 9,024 particles for subsequent processing. An initial map was generated by *ab initio* reconstruction using CryoSPARC (Punjani et al., 2017). These 2D classes were used as references for automatic particle picking from the Titan Krios dataset. The picked particles were extracted at 256^2^ pixel box. The following 2D classification yielded 134,387 particles for subsequent processing. The initial map generated from the Polara dataset was low-pass-filtered to 60 Å and used as a model for preliminary refinement. The resulting particles were 3D classified into six classes. A total of 56,509 particles from the 3D classes containing the whole map of CBF3CCΔN + Ndc10 D1-2 complex were selected to perform a 3D auto-refinement, with the best 3D class low-pass-filtered into 60 Å as reference. After post-processing and map sharpening by the automatically estimated B factor, a 4.2 Å reconstruction was obtained. A mask encompassing the Ndc10 subunit Skp1 and Ctf13 was used for focused refinement of a subset of 22,965 particles to yield a 4.4 Å reconstruction. Local resolution was estimated using RELION.

### Model Building

Cep3 (Purvis and Singleton, 2008) and the BTB/POZ domain of Skp1 (Orlicky et al., 2003) were placed in the CBF3CCΔN map using Chimera (Pettersen et al., 2004). Ctf13 was built *de novo* using Coot (Emsley et al., 2010) in an early 4 Å map, using secondary structure predictions from both Psipred (Jones, 1999) and Phyre2 (Kelley et al., 2015), sequence conservation between common ascomycetes, and with reference to structural preferences for LRR domains (Bella et al., 2008). Tracing of the main chain was assisted using a 6 Å-filtered map. Broken density frequently indicated flexible loops, some of which could be built. Sequence alignments were generated using ClustalW (Larkin et al., 2007) and annotated using ESPript (Gouet et al., 2003). A single round of Real Space Refinement in Phenix was used to refine geometry (Afonine et al., 2012). This model was then rigid body-fit into the final map at 3.7 Å, followed by a final polish in Coot and several rounds of refinement in Phenix and Refmac. Secondary structure restraints were initially generated using the Cep3 and Skp1 crystal structures and, for Ctf13, from within Phenix, with manual editing where deviations from the crystal structures were evident. In order to validate the model, FSCfree and FSCwork were calculated. Using Phenix, the atomic coordinates of the final model were randomly shifted by 0.5 Å and subsequently real space-refined against one unmasked half map (the “working” map). The resulting model was converted to a map and an FSC calculated between it and both the working map (FSCwork) and the free half map (FSCfree).

The atomic model of CBF3CCΔN and the crystal structure of *S. cerevisiae* Ndc10 (PDB: 4ACO) were placed into the CBF3CCΔN plus Ndc10 D1-2 map using Chimera. DNA was modeled using 3D-DART (van Dijk and Bonvin, 2009).

### Gel EMSAs

Protein-DNA interactions were evaluated using EMSA. Twenty-four picomoles singly or doubly labeled 33 bp CDEIII dsDNA (AATATTAGTGTATTTGATTTCCGAAAGTTAAA) was mixed with different amounts of protein with the indicated ratio of DNA to protein in the reaction buffer (25 mM HEPES [pH 8.0], 200 mM KCl, 2 mM DTT, 10% glycerol, 0.02% NP-40, 10 mM MgCl_2_, and 10 μM ZnCl_2_). For competition EMSAs, the unlabeled competitor DNA was 50 times more concentrated than the labeled DNA. The mixtures were incubated at room temperature for 45 min and resolved on a 3%–12% Bis-Tris native polyacrylamide gel at a constant voltage of 150 V at 4°C in 1× native PAGE running buffer for 110 min. After electrophoresis, the gel was scanned using an FLA-3000 fluorescent image analyzer (Fujifilm) excited with a 473 nm laser. EMSAs are representative of at least two repeats, with the exception of those in the Supplemental Information, which were carried out once.

### Protein Stability Assay

The stability of the WT and mutant CBF3CCΔN was assessed using a fluorescence thermal shift assay. The melting temperature of 5 μM protein was measured in a 25 μL volume containing SYPRO orange (Molecular Probes) in 96-well format using a real-time PCR detection system (Mlynek et al., 2014) after buffer exchange into 15 mM HEPES (pH 8.0), 200 mM NaCl, and 2 mM DTT. The melting temperature was determined by non-linear regression analysis of the raw data in GraphPad Prism.

## Data and Software Availability

The accession numbers of the EM maps and atomic coordinates reported in this paper are EMDB: EMD-4241, EMD-0051, EMD-0052 and PDB: 6FE8 and 6GSA.

## Supplemental Information

Document S1. Figures S1–S8 and Tables S1 and S2

Document S2. Article plus Supplemental Information

## References

[bib1] Afonine P.V., Grosse-Kunstleve R.W., Echols N., Headd J.J., Moriarty N.W., Mustyakimov M., Terwilliger T.C., Urzhumtsev A., Zwart P.H., Adams P.D. (2012). Towards automated crystallographic structure refinement with phenix.refine. Acta Crystallogr. D Biol. Crystallogr..

[bib2] Bella J., Hindle K.L., McEwan P.A., Lovell S.C. (2008). The leucine-rich repeat structure. Cell. Mol. Life Sci..

[bib3] Bellizzi J.J., Sorger P.K., Harrison S.C. (2007). Crystal structure of the yeast inner kinetochore subunit Cep3p. Structure.

[bib4] Bui M., Dimitriadis E.K., Hoischen C., An E., Quénet D., Giebe S., Nita-Lazar A., Diekmann S., Dalal Y. (2012). Cell-cycle-dependent structural transitions in the human CENP-A nucleosome in vivo. Cell.

[bib5] Camahort R., Li B., Florens L., Swanson S.K., Washburn M.P., Gerton J.L. (2007). Scm3 is essential to recruit the histone h3 variant cse4 to centromeres and to maintain a functional kinetochore. Mol. Cell.

[bib6] Cho U.S., Harrison S.C. (2011). Ndc10 is a platform for inner kinetochore assembly in budding yeast. Nat. Struct. Mol. Biol..

[bib7] Cole H.A., Howard B.H., Clark D.J. (2011). The centromeric nucleosome of budding yeast is perfectly positioned and covers the entire centromere. Proc. Natl. Acad. Sci. U S A.

[bib8] Cumberledge S., Carbon J. (1987). Mutational analysis of meiotic and mitotic centromere function in Saccharomyces cerevisiae. Genetics.

[bib9] Dalal Y., Wang H., Lindsay S., Henikoff S. (2007). Tetrameric structure of centromeric nucleosomes in interphase Drosophila cells. PLoS Biol..

[bib10] Díaz-Ingelmo O., Martínez-García B., Segura J., Valdés A., Roca J. (2015). DNA topology and global architecture of point centromeres. Cell Rep..

[bib11] Dimitriadis E.K., Weber C., Gill R.K., Diekmann S., Dalal Y. (2010). Tetrameric organization of vertebrate centromeric nucleosomes. Proc. Natl. Acad. Sci. U S A.

[bib12] Emsley P., Lohkamp B., Scott W.G., Cowtan K. (2010). Features and development of Coot. Acta Crystallogr. D Biol. Crystallogr..

[bib13] Espelin C.W., Kaplan K.B., Sorger P.K. (1997). Probing the architecture of a simple kinetochore using DNA-protein crosslinking. J. Cell Biol..

[bib14] Espelin C.W., Simons K.T., Harrison S.C., Sorger P.K. (2003). Binding of the essential Saccharomyces cerevisiae kinetochore protein Ndc10p to CDEII. Mol. Biol. Cell.

[bib15] Fang J., Hogan G.J., Liang G., Lieb J.D., Zhang Y. (2007). The Saccharomyces cerevisiae histone demethylase Jhd1 fine-tunes the distribution of H3K36me2. Mol. Cell. Biol..

[bib16] Fitzgerald-Hayes M., Clarke L., Carbon J. (1982). Nucleotide sequence comparisons and functional analysis of yeast centromere DNAs. Cell.

[bib17] Furuyama S., Biggins S. (2007). Centromere identity is specified by a single centromeric nucleosome in budding yeast. Proc. Natl. Acad. Sci. U S A.

[bib18] Furuyama T., Henikoff S. (2009). Centromeric nucleosomes induce positive DNA supercoils. Cell.

[bib19] Gaudet A., Fitzgerald-Hayes M. (1987). Alterations in the adenine-plus-thymine-rich region of CEN3 affect centromere function in Saccharomyces cerevisiae. Mol. Cell. Biol..

[bib20] Gouet P., Robert X., Courcelle E. (2003). ESPript/ENDscript: extracting and rendering sequence and 3D information from atomic structures of proteins. Nucleic Acids Res..

[bib21] Han X.-R., Zha Z., Yuan H.-X., Feng X., Xia Y.-K., Lei Q.-Y., Guan K.-L., Xiong Y. (2016). KDM2B/FBXL10 targets c-Fos for ubiquitylation and degradation in response to mitogenic stimulation. Oncogene.

[bib22] Hegemann J.H., Shero J.H., Cottarel G., Philippsen P., Hieter P. (1988). Mutational analysis of centromere DNA from chromosome VI of Saccharomyces cerevisiae. Mol. Cell. Biol..

[bib23] Ho K.-H., Tsuchiya D., Oliger A.C., Lacefield S. (2014). Localization and function of budding yeast CENP-A depends upon kinetochore protein interactions and is independent of canonical centromere sequence. Cell Rep..

[bib24] Jehn B., Niedenthal R., Hegemann J.H. (1991). In vivo analysis of the Saccharomyces cerevisiae centromere CDEIII sequence: requirements for mitotic chromosome segregation. Mol. Cell. Biol..

[bib25] Jones D.T. (1999). Protein secondary structure prediction based on position-specific scoring matrices. J. Mol. Biol..

[bib26] Kelley L.A., Mezulis S., Yates C.M., Wass M.N., Sternberg M.J. (2015). The Phyre2 web portal for protein modeling, prediction and analysis. Nat. Protoc..

[bib27] Kim T., Buratowski S. (2007). Two Saccharomyces cerevisiae JmjC domain proteins demethylate histone H3 Lys36 in transcribed regions to promote elongation. J. Biol. Chem..

[bib28] Kimanius D., Forsberg B.O., Scheres S.H.W., Lindahl E. (2016). Accelerated cryo-EM structure determination with parallelisation using GPUs in RELION-2. eLife.

[bib29] Klose R.J., Kallin E.M., Zhang Y. (2006). JmjC-domain-containing proteins and histone demethylation. Nat. Rev. Genet..

[bib30] Krassovsky K., Henikoff J.G., Henikoff S. (2012). Tripartite organization of centromeric chromatin in budding yeast. Proc. Natl. Acad. Sci. U S A.

[bib31] Kwon D.-W., Ahn S.H. (2011). Role of yeast JmjC-domain containing histone demethylases in actively transcribed regions. Biochem. Biophys. Res. Commun..

[bib32] Larkin M.A., Blackshields G., Brown N.P., Chenna R., McGettigan P.A., McWilliam H., Valentin F., Wallace I.M., Wilm A., Lopez R. (2007). Clustal W and Clustal X version 2.0. Bioinformatics.

[bib33] Leber V., Nans A., Singleton M.R. (2018). Structural basis for assembly of the CBF3 kinetochore complex. EMBO J..

[bib34] Lechner J. (1994). A zinc finger protein, essential for chromosome segregation, constitutes a putative DNA binding subunit of the Saccharomyces cerevisiae kinetochore complex, Cbf3. EMBO J..

[bib35] Lechner J., Carbon J. (1991). A 240 kd multisubunit protein complex, CBF3, is a major component of the budding yeast centromere. Cell.

[bib36] MacPherson S., Larochelle M., Turcotte B. (2006). A fungal family of transcriptional regulators: the zinc cluster proteins. Microbiol. Mol. Biol. Rev..

[bib37] Malik H.S., Henikoff S. (2009). Major evolutionary transitions in centromere complexity. Cell.

[bib38] Marmorstein R., Carey M., Ptashne M., Harrison S.C. (1992). DNA recognition by GAL4: structure of a protein-DNA complex. Nature.

[bib39] McGrew J., Diehl B., Fitzgerald-Hayes M. (1986). Single base-pair mutations in centromere element III cause aberrant chromosome segregation in Saccharomyces cerevisiae. Mol. Cell. Biol..

[bib40] Mizuguchi G., Xiao H., Wisniewski J., Smith M.M., Wu C. (2007). Nonhistone Scm3 and histones CenH3-H4 assemble the core of centromere-specific nucleosomes. Cell.

[bib41] Mlynek G., Lehner A., Neuhold J., Leeb S., Kostan J., Charnagalov A., Stolt-Bergner P., Djinović-Carugo K., Pinotsis N. (2014). The Center for Optimized Structural Studies (COSS) platform for automation in cloning, expression, and purification of single proteins and protein-protein complexes. Amino Acids.

[bib42] Ng R., Carbon J. (1987). Mutational and in vitro protein-binding studies on centromere DNA from Saccharomyces cerevisiae. Mol. Cell. Biol..

[bib43] Niedenthal R., Stoll R., Hegemann J.H. (1991). In vivo characterization of the Saccharomyces cerevisiae centromere DNA element I, a binding site for the helix-loop-helix protein CPF1. Mol. Cell. Biol..

[bib44] Orlicky S., Tang X., Willems A., Tyers M., Sicheri F. (2003). Structural basis for phosphodependent substrate selection and orientation by the SCFCdc4 ubiquitin ligase. Cell.

[bib45] Perriches T., Singleton M.R. (2012). Structure of yeast kinetochore Ndc10 DNA-binding domain reveals unexpected evolutionary relationship to tyrosine recombinases. J. Biol. Chem..

[bib46] Pettersen E.F., Goddard T.D., Huang C.C., Couch G.S., Greenblatt D.M., Meng E.C., Ferrin T.E. (2004). UCSF Chimera—a visualization system for exploratory research and analysis. J. Comput. Chem..

[bib47] Pietrasanta L.I., Thrower D., Hsieh W., Rao S., Stemmann O., Lechner J., Carbon J., Hansma H. (1999). Probing the Saccharomyces cerevisiae centromeric DNA (CEN DNA)-binding factor 3 (CBF3) kinetochore complex by using atomic force microscopy. Proc. Natl. Acad. Sci. U S A.

[bib48] Punjani A., Rubinstein J.L., Fleet D.J., Brubaker M.A. (2017). cryoSPARC: algorithms for rapid unsupervised cryo-EM structure determination. Nat. Methods.

[bib49] Purvis A., Singleton M.R. (2008). Insights into kinetochore-DNA interactions from the structure of Cep3Δ. EMBO Rep..

[bib50] Rodrigo-Brenni M.C., Thomas S., Bouck D.C., Kaplan K.B. (2004). Sgt1p and Skp1p modulate the assembly and turnover of CBF3 complexes required for proper kinetochore function. Mol. Biol. Cell.

[bib51] Rohou A., Grigorieff N. (2015). CTFFIND4: Fast and accurate defocus estimation from electron micrographs. J. Struct. Biol..

[bib52] Rosenthal P.B., Henderson R. (2003). Optimal determination of particle orientation, absolute hand, and contrast loss in single-particle electron cryomicroscopy. J. Mol. Biol..

[bib53] Russell I.D., Grancell A.S., Sorger P.K. (1999). The unstable F-box protein p58-Ctf13 forms the structural core of the CBF3 kinetochore complex. J. Cell Biol..

[bib54] Scheres S.H.W., Chen S. (2012). Prevention of overfitting in cryo-EM structure determination. Nat. Methods.

[bib55] Sein H., Värv S., Kristjuhan A. (2015). Distribution and maintenance of histone H3 lysine 36 trimethylation in transcribed locus. PLoS ONE.

[bib56] Shaikh T.R., Gao H., Baxter W.T., Asturias F.J., Boisset N., Leith A., Frank J. (2008). SPIDER image processing for single-particle reconstruction of biological macromolecules from electron micrographs. Nat. Protoc..

[bib57] Sheard L.B., Tan X., Mao H., Withers J., Ben-Nissan G., Hinds T.R., Kobayashi Y., Hsu F.-F., Sharon M., Browse J. (2010). Jasmonate perception by inositol-phosphate-potentiated COI1-JAZ co-receptor. Nature.

[bib58] Shivaraju M., Unruh J.R., Slaughter B.D., Mattingly M., Berman J., Gerton J.L. (2012). Cell-cycle-coupled structural oscillation of centromeric nucleosomes in yeast. Cell.

[bib59] Tan X., Calderon-Villalobos L.I.A., Sharon M., Zheng C., Robinson C.V., Estelle M., Zheng N. (2007). Mechanism of auxin perception by the TIR1 ubiquitin ligase. Nature.

[bib60] Tolstorukov M.Y., Colasanti A.V., McCandlish D.M., Olson W.K., Zhurkin V.B. (2007). A novel roll-and-slide mechanism of DNA folding in chromatin: implications for nucleosome positioning. J. Mol. Biol..

[bib61] Trewick S.C., Minc E., Antonelli R., Urano T., Allshire R.C. (2007). The JmjC domain protein Epe1 prevents unregulated assembly and disassembly of heterochromatin. EMBO J..

[bib62] Urnavicius L., Zhang K., Diamant A.G., Motz C., Schlager M.A., Yu M., Patel N.A., Robinson C.V., Carter A.P. (2015). The structure of the dynactin complex and its interaction with dynein. Science.

[bib63] van Dijk M., Bonvin A.M.J.J. (2009). 3D-DART: a DNA structure modelling server. Nucleic Acids Res..

[bib64] Venkatesh S., Smolle M., Li H., Gogol M.M., Saint M., Kumar S., Natarajan K., Workman J.L. (2012). Set2 methylation of histone H3 lysine 36 suppresses histone exchange on transcribed genes. Nature.

[bib65] Verdaasdonk J.S., Bloom K. (2011). Centromeres: unique chromatin structures that drive chromosome segregation. Nat. Rev. Mol. Cell Biol..

[bib66] Wong S.J., Gearhart M.D., Taylor A.B., Nanyes D.R., Ha D.J., Robinson A.K., Artigas J.A., Lee O.J., Demeler B., Hart P.J. (2016). KDM2B recruitment of the polycomb group complex, PRC1.1, requires cooperation between PCGF1 and BCORL1. Structure.

[bib67] Zhang W., Colmenares S.U., Karpen G.H. (2012). Assembly of Drosophila centromeric nucleosomes requires CID dimerization. Mol. Cell.

[bib68] Zheng S.Q., Palovcak E., Armache J.-P., Verba K.A., Cheng Y., Agard D.A. (2017). MotionCor2: anisotropic correction of beam-induced motion for improved cryo-electron microscopy. Nat. Methods.

