# Insights into Centromere DNA Bending Revealed by the Cryo-EM Structure of the Core Centromere Binding Factor 3 with Ndc10

**DOI:** 10.1016/j.celrep.2019.01.026

**Published:** 2019-01-22

**Authors:** Wenjuan Zhang, Natalya Lukoyanova, Shomon Miah, Jonathan Lucas, Cara K. Vaughan

(Cell Reports *24*, 744–754; July 17, 2018)

In the originally published version of this article, author name Natalya Lukoyanova was misspelled in the author list. This has now been corrected.

The authors regret this error.

