# Supplementary material for: Insights into Centromere DNA Bending Revealed by the Cryo-EM Structure of the Core Centromere Binding Factor 3 with Ndc10
Source: Cell Rep. 2018 Jul 17;24(3):744–54. doi: 10.1016/j.celrep.2018.06.068 (PMC6077249; doi:10.1016/j.celrep.2018.06.068)
Supplement: Document S1. Figures S1–S8 and Tables S1 and S2 [file mmc1.pdf]

Cell Reports, Volume *24*

Supplemental Information

Insights into Centromere DNA Bending Revealed by the Cryo-EM Structure of the Core Centromere Binding Factor 3 with Ndc10

Wenjuan Zhang, Natalya Lukoynova, Shomon Miah, Jonathan Lucas, and Cara K. Vaughan

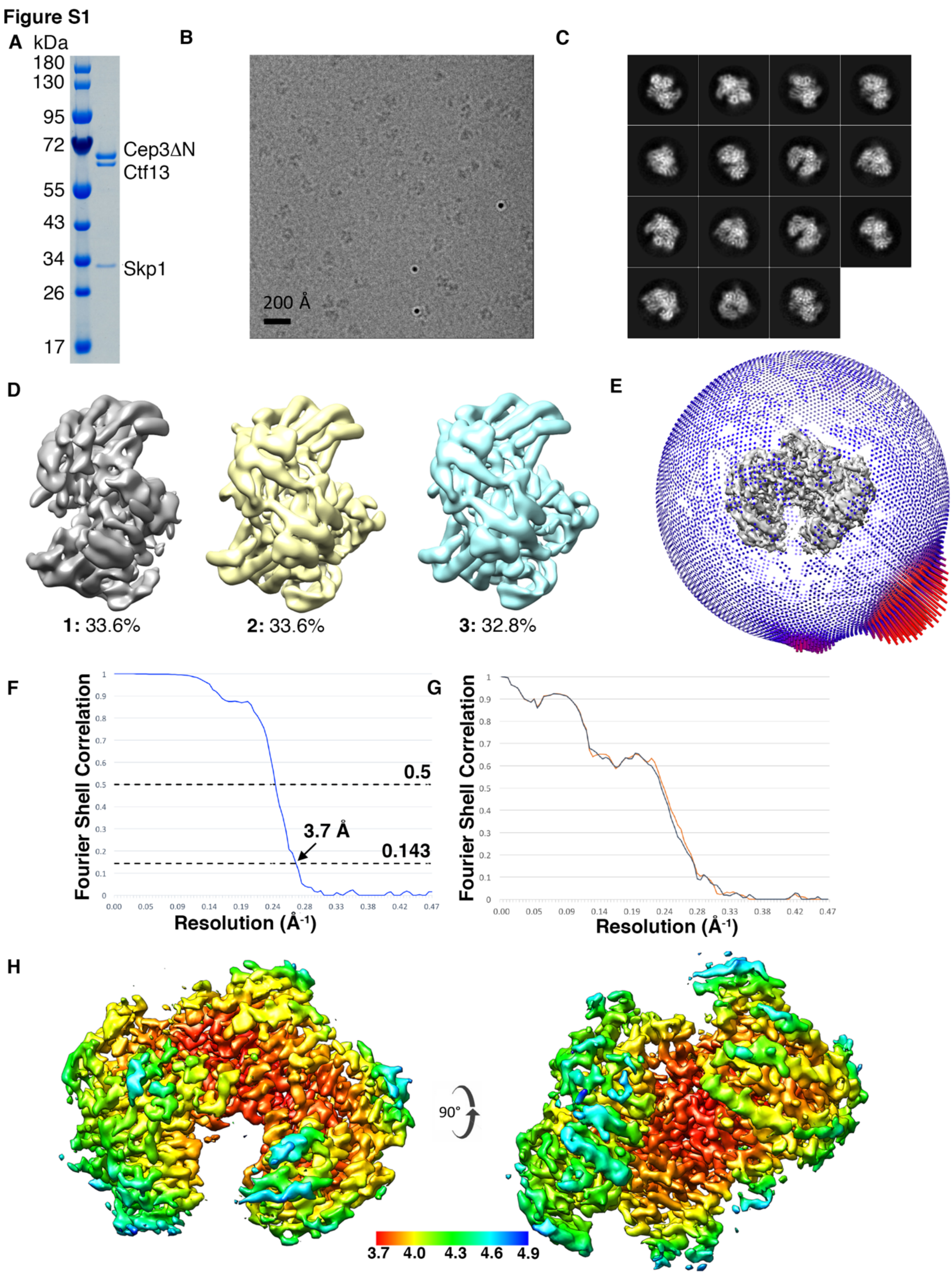

Figure S1: Cryo-EM structure determination and analysis for CBF3CCΔN. Related to Figures 1 and 2. (A) SDS-PAGE of purified CBFCCΔN. (B) Representative cryo-EM micrograph. (C) Representative 2D class averages. (D) Output from 3D classification in Relion. Model 2 was

selected for further refinement. (E) Angular distribution of particles contributing to the final map. (F) ‘Gold standard’ fourier shell correlation between the final masked half maps showing a resolution of 3.7 Å. (G) Model to map fourier shell correlation for the working and free final half maps. (H) Map of CBF3CCΔN coloured by local resolution. Scale bar shows the resolution in Ångströms.

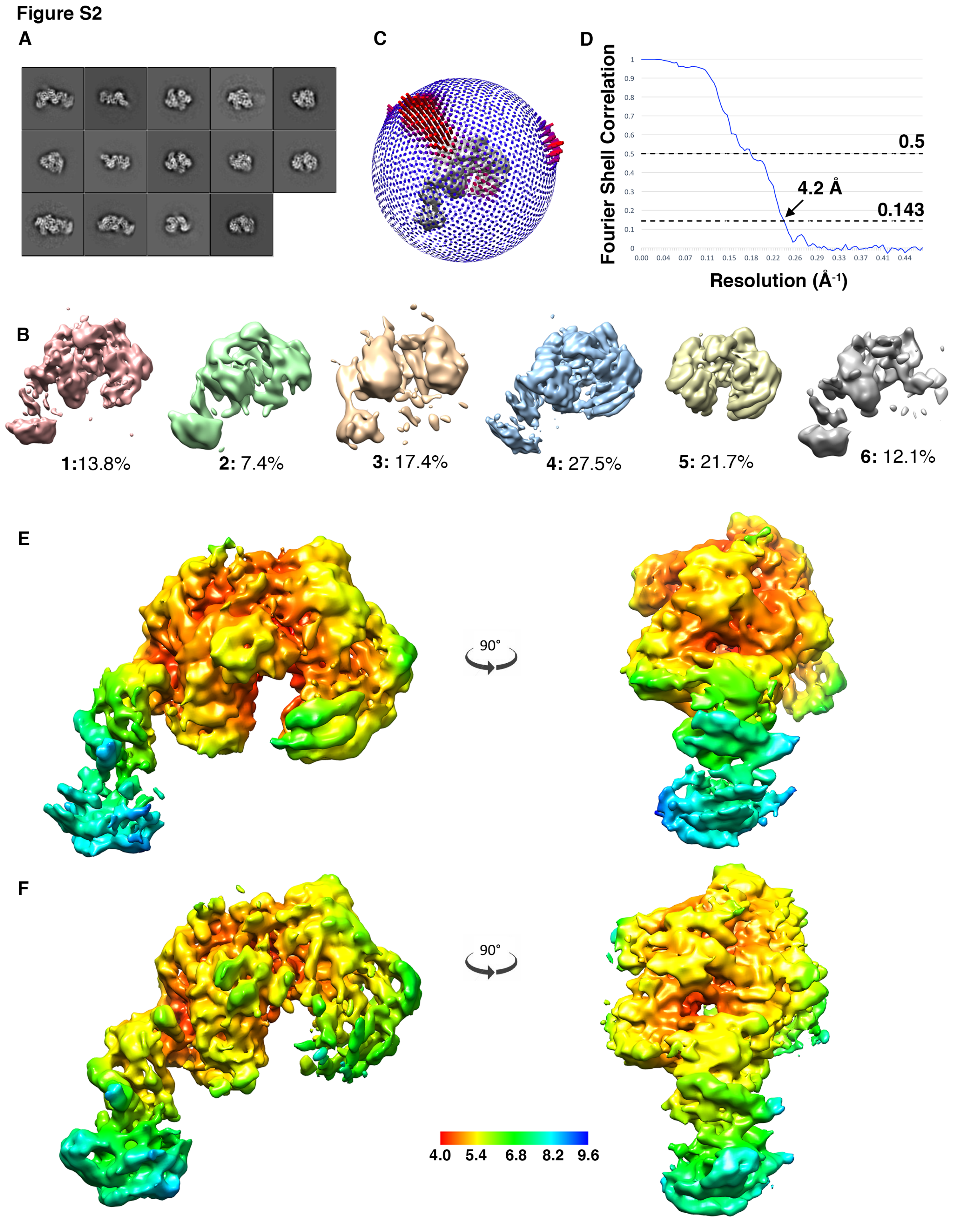

Figure S2: Cryo-EM structure determination and analysis for CBF3CCΔN + Ndc10 D1-2. Related to Figures 1 and 5. (A) Representative 2D classes. (B) Output from 3D classification. Models 1 & 4 were selected for further refinement. (C) Angular distribution of particles

contributing to the final map. (D) ‘Gold standard’ fourier shell correlation between the final masked half maps showing a resolution 4.2 Å. (E) Full map and (F) map generated by focused refinement on Ndc10 D1-2 coloured by local resolution. The scale bar shows the resolution in Ångströms.

Figure S3

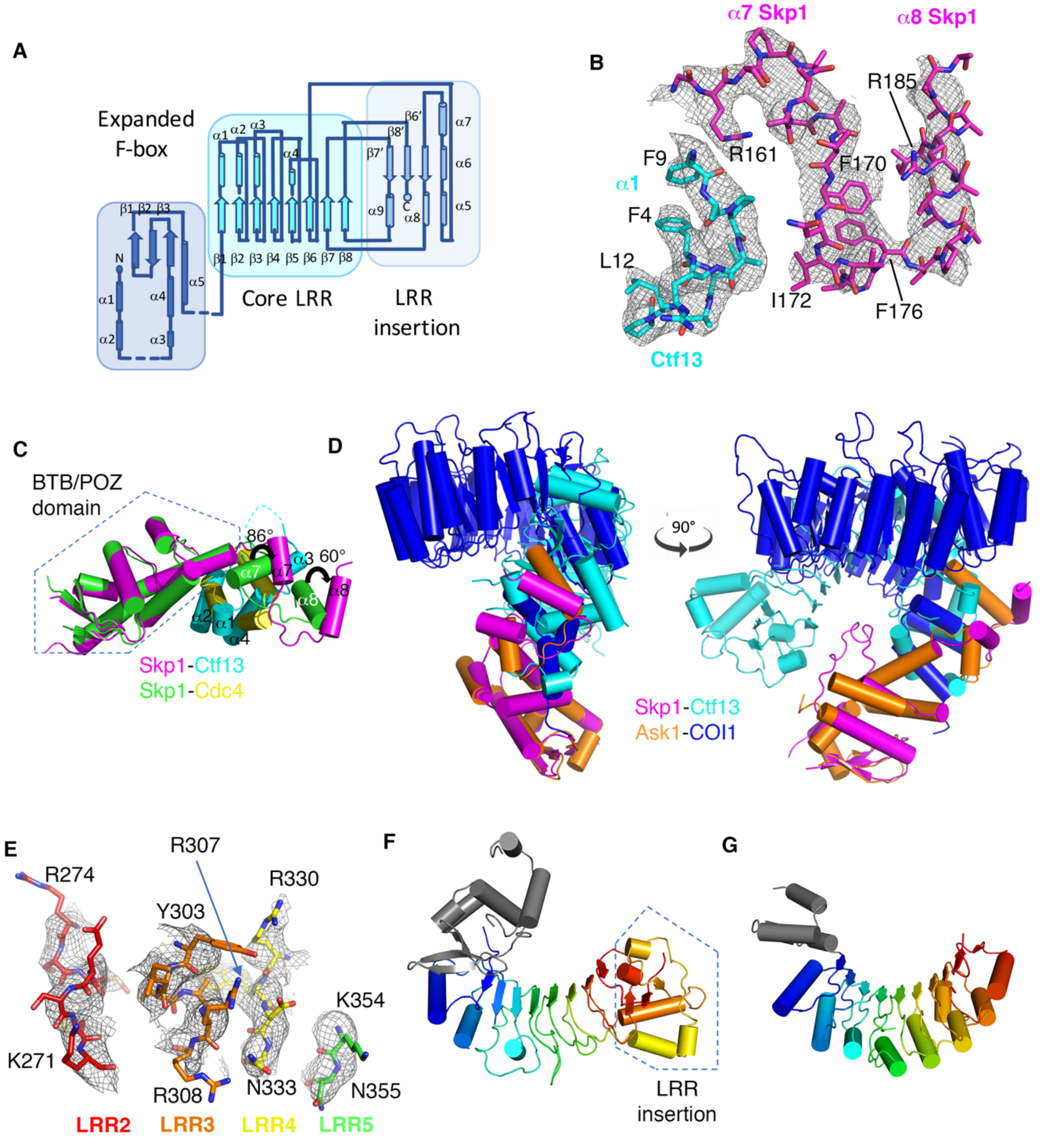

Figure S3: Skp1-Ctf13 forms an atypical Skp1-FBXL complex. Related to Figures 2 and 3. (A) Topology diagram for Ctf13. Dashed lines represent parts of the structure for which no electron density was observed. (B) Electron density map for helices α7 and α8 from Skp1

(magenta) and the N-terminal residues from Ctf13 F-box (cyan). Other helices are omitted for clarity. (C) An RMSD fit of Skp1 from CBF3CCΔN with Skp1 from the *S. cerevisiae* Skp1-Cdc4 complex (PDB 3V7D) which adopts the canonical Skp1 F-box conformation. Skp1-Ctf13$^{F\text{-}box}$ are in magenta and cyan; Skp1-Cdc4$^{F\text{-}box}$ are in green and yellow. The BTB/POZ domain of Skp1 is boxed. The change in angles of the $\alpha 7$ and $\alpha 8$ helices between each Skp1 structure are shown. (D) An RMSD fit of Skp1 from Skp1-Ctf13 (magenta-cyan) with the Arabidopsis Skp1 homologue, Ask1, from Ask1-COI1 (orange-blue) (PDB 3OGK). Skp1-Ctf13 are in the same plane, whereas the LRR of COI1 is almost perpendicular to the Skp1-Fbox domains. (E) Electron density and refined atomic model highlighting the basic residues of Ctf13 that project from the LRR motifs lining the channel. (F) Cartoon representations of Ctf13 and (G) its closest structural homologue containing an F-box, human KDM2B (PDB 5JH5). The LRR domains are coloured rainbow and the F-box is coloured grey. The LRR insertion is highlighted in a dashed box.

Figure S4

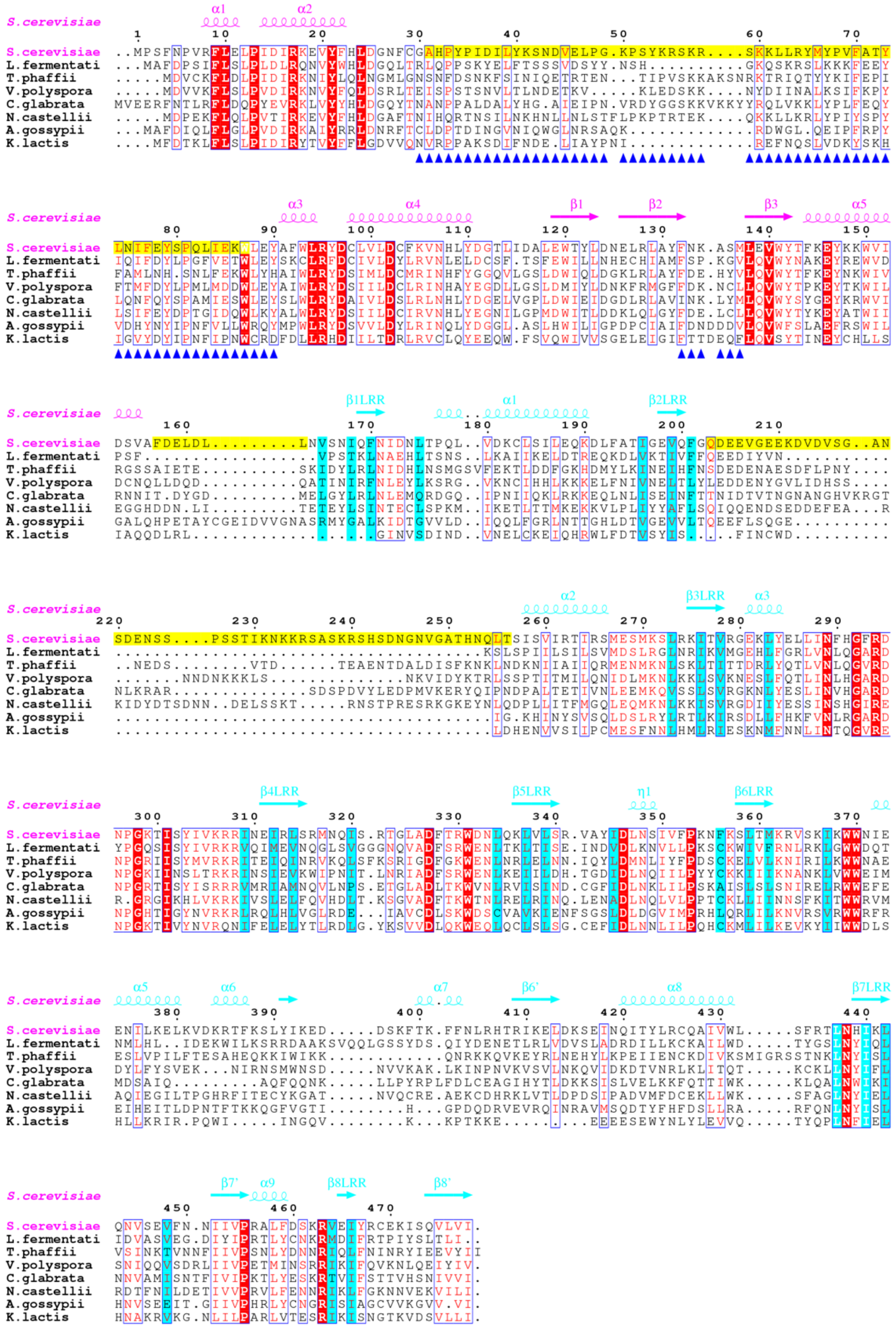

Figure S4: Sequence alignment of Ctf13 homologues annotated with secondary structure.

Related to Figure 2. Sequence alignment of Ctf13 homologues annotated above in magenta with the secondary structure of the expanded F-box, and in cyan for the secondary structure

of the LRR domain. Residues in consensus hydrophobic positions in each LRR motif are highlighted in cyan. Residues not visible in the electron density map for the core complex are highlighted yellow and residues that form the interface with Ndc10 are marked with blue triangles.

Figure S5

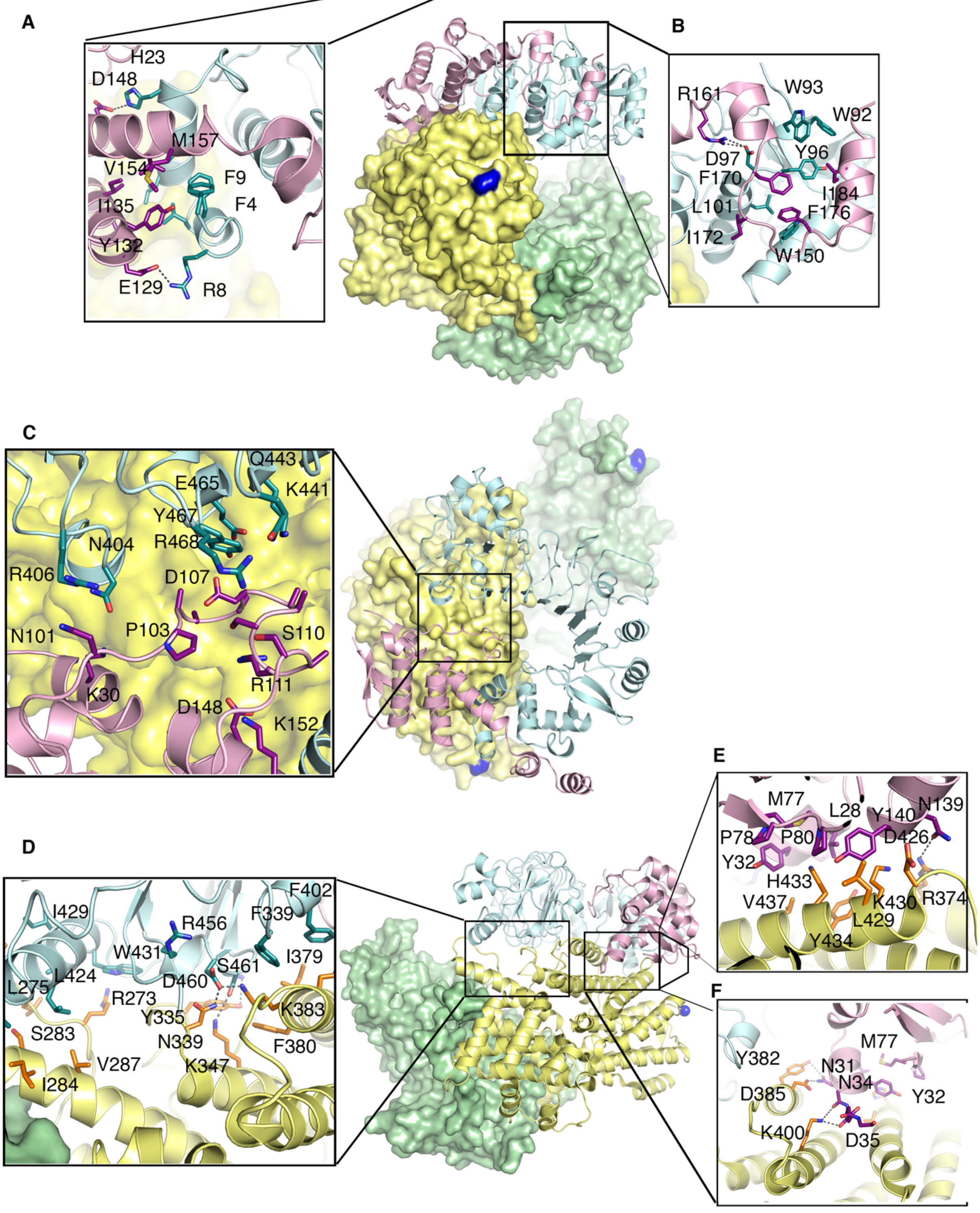

Figure S5: Detailed analysis of the interfaces within CBF3CCΔN. Related to Figure 2.

Views showing the interactions contributing to (A & B) the Skp1-Ctf13 F-box interface, (C)

Skp1 with the LRR insertion of Ctf13, (D) the Cep3-Ctf13 interface and (E & F) the Cep3-Skp1 interface.

Figure S6

A

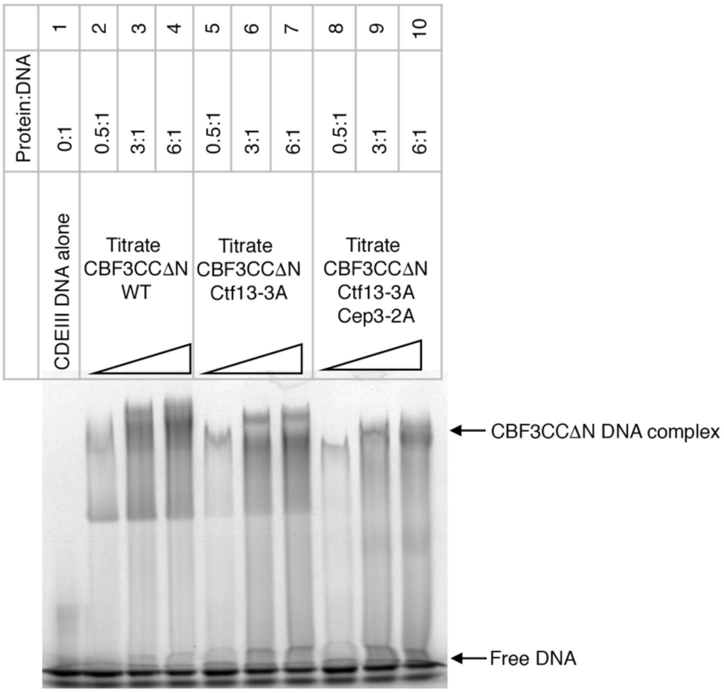

B

CBF3CCΔN	Mono Q conductivity at elution (mS/cm)	Analytical S200 elution volume (ml)	T_m (°C)
Wild-type	20.6	11.5	49.66 ± 0.05
Ctf13-AAA	20.7	11.1	49.34 ± 0.08
Ctf13-AAA, Cep3-AA	22.35	10.7	43.52 ± 0.30

Figure S6: DNA-binding and biophysical properties of mutants of CBF3CCΔN. Related to Figure 4. (A) Electrophoretic mobility shift assays performed with 1.6 μM fluorescently-labelled CDEIII DNA and a titration of CBF3CCΔN (lanes 2-4), CBF3CCΔN with Ctf13 mutations R307A-R308A-R330A (lanes 5-7), and CBF3CCΔN with both Ctf13 R307A-R308A-R330A and Cep3 K265A-K364A. (B) Biophysical characterisation of wild-type CBF3CCΔN, and mutant complexes containing Ctf13 R307A-R308A-R330A (Ctf13AAA), and Ctf13 R307A-R308A-R330A + Cep3 K265A-K364A (Ctf13-AAA, Cep3-AA): conductivity at peak elution on Mono Q, elution volume on S200 Increase, and melting temperature (T_m) are reported.

Figure S7

A

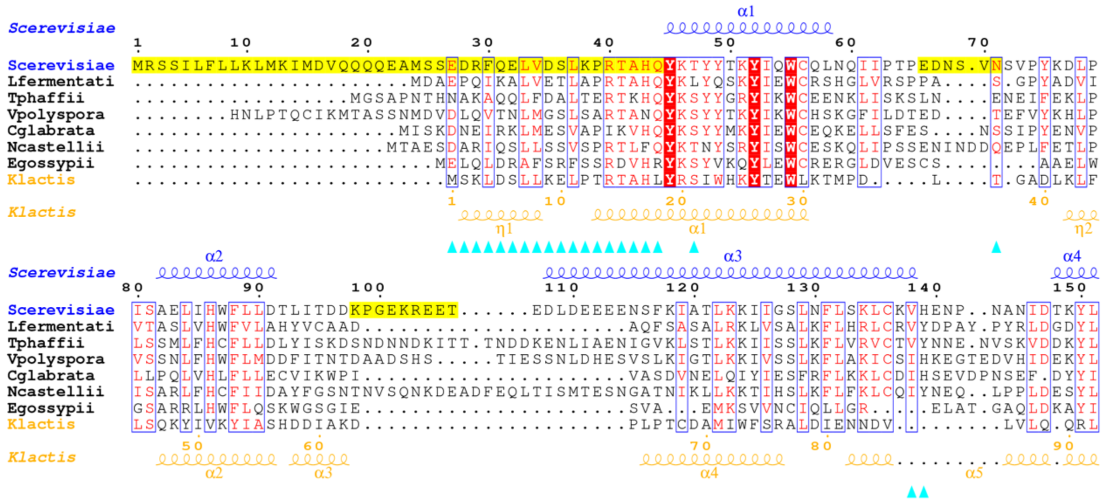

B

	1	2	3	4	5	6	7	8	9	10
Protein:DNA	0:1	1:1	1:2	1:4	1:8	1:16	1:0	1:0	1:1	1:1:1
CBF3CCΔN	-	+	+	+	+	+	+	-	-	+
Ndc10 D1-2	-	-	-	-	-	-	-	+	+	+

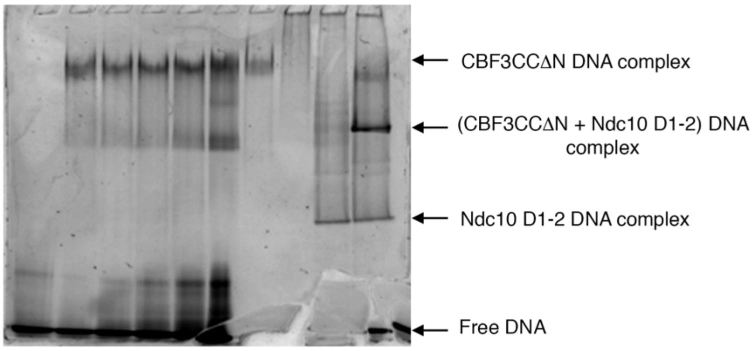

Figure S7: Sequence alignment and DNA-binding analysis for Ndc10 D1-2. Related to Figure 5. (A) Sequence alignment for the first ~150 residues of Ndc10 homologues annotated by the secondary structure of the *S.cerevisiae* protein (PDB 4ACO) (blue) and the *K. lactis* protein (PDB 3SQI) (orange). Residues not visible in 4ACO are highlighted in yellow. Residues found at the interface with Ctf13 are highlighted with cyan triangles.

(B) Electrophoretic mobility shift assays performed with 3.2 μM CBF3CCΔN and unlabelled 33 bp CDEIII, plus equimolar concentration of Ndc10 D1-2 and visualised using SyBr stain. CBF3CCΔN binds SyBr stain non-specifically (lane 7).

Figure S8

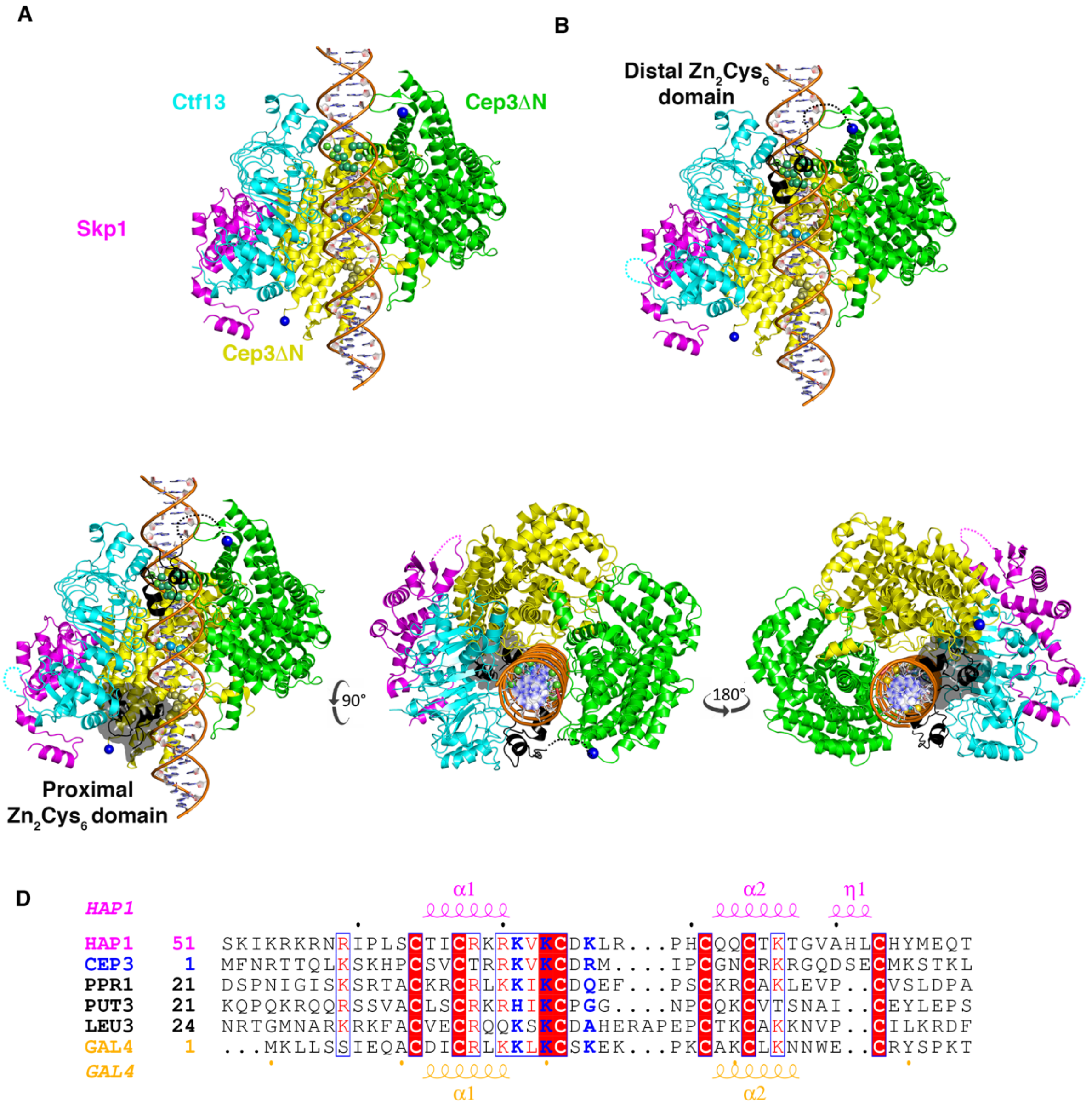

Figure S8: Modelling of CDEIII binding to CBF3CCΔN. Related to Figure 6. (A) Linear CEN3 CDEIII DNA, modelled using 3D-DART (van Dijk and Bonvin, 2009), aligned in the channel as described in the main text. (B) The Zn_2Cys_6 domain from GAL4 (PDB 1D66) is used to model the Cep3 domain. Superposition of this domain (black cartoon) on the CCG is readily accommodated between Ctf13 (cyan) and one protomer of Cep3 (green). (C) A similar superposition on the TGT half-site results in a steric clash of the Zn_2Cys_6 domain (black cartoon with transparent surface) with Ctf13. (D) Sequence alignment of the Zn_2Cys_6 domain

of Cep3 with GAL4-like fungal transcription factors of known structure, annotated above and below the alignment with the secondary structure for two representative members. Residues whose side chains contribute to half-site CCG binding are highlighted in blue.

Table S1: Statistics for data collection, processing and final maps. Related to Figure 1.

	CBF3CCΔN dataset 1	***CBF3CCΔN dataset 2***	***CC+Ndc10 D1-2***	
**Data collection**				
Microscope	FEI Titan Krios			
Voltage (kV)	300			
Electron dose (e Å^{-2})	46	60.9	50	
Detector	Gatan K2 Summit			
Pixel size (Å)	1.06	1.06	1.06	
Defocus range (μm)	-1.6 to -3.7	-1.0 to -4.0	-1.5 to -3.5	
Particles picked by template matching after 2D classification	69392	212724	139102	
**Reconstruction (RELION)**				focused refinement
Particles	187606		56509	22965
Box size (pixels)	200		256	256
Accuracy of rotations (°)	1.87		2.63	3.16
Accuracy of translations (pixels)	0.73		1.29	1.27
Map-sharpening *B* factor (Å^2)	-167		-2.0	-6.5
Final resolution (Å)	3.7		4.2	4.4

Table S2: Statistics for CBF3CCΔN atomic model. Related to Figure 2.

**Model composition**	
Map CC (around atoms)	0.78
Protein residues	1547
**Refinement**	
Resolution (Å)	3.7
$FSC_{average}$	0.85
**R.m.s. deviation**	
Bond length (Å)	0.004
Bond angle (°)	0.864
**Validation**	
Clashscore, all atoms	4.16
Rotamer outliers (%)	0.45
**Ramachandran plot**	
Favored (%)	94.42
Allowed (%)	5.52
Outliers (%)	0.07
**Disordered regions**	
Cep3A	321-329; 566-587
Cep3B	318,329; 566-587
Skp1	39-74
Ctf13	31-87; 157-163; 203-256
**Side chains fitted of ordered mainchain (%)**	
Cep3 chain A	98.3
Cep3 chain B	99.4
Skp1	85.3
Ctf13	80.6
